# Household environmental tobacco smoke exposure in healthy young children in Hong Kong: Prevalence and risk factors

**DOI:** 10.1371/journal.pone.0227733

**Published:** 2020-01-14

**Authors:** Siyu Dai, Kate Ching Ching Chan

**Affiliations:** Department of Paediatrics, Faculty of Medicine, The Chinese University of Hong Kong, Hong Kong SAR, China; University of South Florida, UNITED STATES

## Abstract

**Background:**

Environmental tobacco smoke (ETS) exposure attributable respiratory illness burden is huge in paediatric population. Understanding the epidemiology of ETS exposure is important to guide health promotion planning. Therefore, we designed this study to determine the prevalence of household ETS exposure in healthy young children under 2 years of age in Hong Kong, and to explore risk factors associated with the exposure. Our secondary goal was to characterise children’s exposure profile to maternal smoking.

**Methods:**

A secondary analysis was performed based on the data collected from our 2013–2014 territory-wide cross-sectional pneumococcal carriage surveillance study, with a sample size of 1541. We conducted descriptive analysis for exposure prevalence, univariate and multivariate analysis for identification of risk factors.

**Results:**

1541 children (mean age: 11.2 ± 6.4 months, male: 50.7%) were included in the analysis. The overall prevalence of current household ETS exposure was 31.5%, prevalence of prenatal and postnatal maternal smoking was 3.5% and 1.6% respectively. Independent factors associated with children’s ETS exposure were: never breastfed (AOR: 1.48, 95% CI: 1.13–1.93, p = 0.004); prenatal maternal smoking (AOR: 7.46, 95% CI: 2.73–20.39, p< 0.001); overcrowding of household living place (AOR: 3.17, 95% CI: 2.02–4.96, P< 0.001); lower household income (AOR: 1.34, 95% CI: 1.04–1.72, p = 0.02). Interestingly, children residing in Kowloon (AOR: 1.66, 95% CI: 1.19–2.33, p = 0.003) and New Territories West (AOR: 1.54, 95% CI: 1.11–2.15, p = 0.01) were associated with exposure compared with children residing in Hong Kong Island.

**Conclusion:**

Exposure to household ETS is prevalent among Hong Kong young children, particularly in children with maternal unfavourable behaviour and lower socioeconomic status. The identified risk factors should be considered while tobacco control interventions and legislations are planned.

## Introduction

ETS is defined as tobacco smoke produced by an active smoker both from the exhalation of smoked tobacco and by the burning end of the cigarette, which is inhaled by non-smokers.[1] There is no safe exposure level of environmental tobacco smoke (ETS) exposure, children and fetus are especially sensitive to the exposure.[1–2] Children’s ETS exposure most commonly takes place in their homes and the main source is parental smoking.[3] The exposure attributable disease burden is huge. Lower respiratory tract infection (LRTI) in children younger than 5 years was reported to be the largest global disease burden of ETS [4], while LRTI is a leading infectious cause of mortality in children younger than 5 years worldwide.[5]

Globally, the prevalence of ETS exposure in children was 40% in year 2004.[4] In Hong Kong, the prevalence was quite similar.[6] For young children and infants, the information is relatively scarce. A previous birth cohort study reported a prevalence of 42.1% in 1997.[7] However, many milestones on local tobacco control have been achieved thereafter, such as the public smoke-free legislation since 2007. According to a recent meta-analysis of the impact of public smoking bans on children’s household ETS exposure, majority of the included studies reported a decrease in the post-legislation exposure prevalence, while two studies including one Hong Kong study reported an increase.[8] *Ho S*. et al. indicated that comprehensive smoke-free legislation without strong support for smoking cessation might have displaced smoking into the homes of school-aged children.[9] However, the condition of young children remained unknown.

A review study shows that having smoking parents, lower family socioeconomic status (SES), lower parental education level, and having less negative parental attitudes towards ETS are shown to be risk factors of exposure.[10] However, majority of the existing publications were done in elder children, only three studies explored the risk factors of ETS exposure among children under two years of age, although younger children can actually be more susceptible to the exposure risks. A Thai study found that smoking in the presence of an infant was associated with paternal age of 25–34 or more than 44 years, low parental education level, and a Muslim father.[11] In Iran, lower social status and younger infant's age were associated with the exposure.[12] More recently, a large-scale American study reported that infants of families having more children and having smoking mother were less likely to have a no-smoke policy and more likely to report that someone smoked inside the home.[13] The household ETS exposure profile has not been comprehensively drawn yet in Hong Kong young children, cultural difference may have significant influence on the epidemiology.

Understanding the epidemiology profile of ETS exposure is important to guide health promotion planning such as to identify at-risk population and to distribute limited resources to decrease ETS attributable disease burden appropriately and efficiently. Therefore, we designed this study to determine the prevalence of household ETS exposure in healthy young children under 2 years of age in Hong Kong, and to explore risk factors associated with exposure. Our secondary goal was to characterise children’s exposure profile to maternal smoking.

## Materials and methods

### Study population

We carried out this secondary analysis based on the data obtained from our 2013–2014 community-based, territory-wide cross-sectional pneumococcal carriage surveillance study. Children aged 2 months, 12 months and 18 months, across 4 main regions of Hong Kong were recruited randomly when they attended the health service provided by the Maternal and Child Health Centre. Details of the surveillance study have been published previously.[14] This retrospective study was approved by the Joint Chinese University of Hong Kong-New Territories East Cluster Clinical Research Ethics Committee (CRE-2018.452).

### Data collection

In-depth interview was carried out in the previous surveillance study. Caregivers of the young children were asked to complete standardised self-administered questionnaire under supervision by a trained research nurse, and the completed questionnaire was checked by our research nurse at the study site. Furthermore, clarifications would be made over the phone by research nurse if necessary. Data on a range of variables including demographics information, children’s clinical characteristics and their household tobacco exposure status were collected. Concerning the household ETS exposure, information about smoking of any household members, prenatal maternal smoking and postnatal maternal smoking were collected.

### Variable measurements

Binary variables on exposure were current household ETS exposure, prenatal maternal smoking exposure and postnatal maternal smoking exposure. The caregiver was asked whether there were any household smokers living with the child (yes vs. no), whether the mother smoked during pregnancy (yes vs. no), and whether the mother was an active smoker (yes vs. no). Exposure level was examined using ordinal categorical variables including number of household smokers at home (0, 1, >1), and daily total cigarettes consumption by all household smokers (0 cigarettes/ day, 1–20 cigarettes/ day, >20 cigarettes/ day). Those potential associated risk factors (demographic and clinical) including children’s age, sex, history of breastfeeding (yes vs. no), having siblings (yes vs. no), living region, overcrowding of living area (a living space of <5.5 m^2^/person in accordance with the guideline of the Hong Kong Housing Authority), low household income (household monthly income ≤HK$20,000). Our measures of variables were similar with previous studies.[7, 15]

### Sample size calculation

Based on previous local and global studies, the estimated prevalence of household ETS exposure in Hong Kong children was 30%.[4, 7] Our study was powered to estimate ETS exposure prevalence of 30% with 80% power with 2.5% possible error, which required a minimum sample size of 1289 (Epi Info V.7.2).[16]

### Statistical method

Period prevalence of children’s household ETS exposure was calculated. As in previous surveillance study, data were not collected at one time, they were collected during Jun 2013 to June 2014. As for the prevalence calculation equation, the denominator is the number of participants in our previous surveillance study, while numerator was the number of children reported to be exposed to each ETS variables. Categorical risk factors of ETS exposure condition were identified by univariate analysis using Chi square test. Variables that were significant in the univariate analysis as defined with a p value< 0.05, and those with a p value< 0.1 and could increase the risk of having household ETS exposure from a clinical point of view were further tested by multivariate logistic regression using the forward-conditional model. Adjustments were carried out to minimise potential confounding effect. Since the effect of prenatal maternal smoking on children’s current household ETS exposure condition could be dominate [17], thus different models were built: 1) by ignoring the effect of prenatal maternal smoking; 2) by adjusting to prenatal maternal smoking; 3) by excluding young children with prenatal maternal smoking. Nagelkerke R-square value which stands for the percent of variance could be explained by the model would be stated. Model with the largest R-square value was further interpreted. A p-value of <0.05 was considered to be statistically significant. All statistical analysis was performed by the SPSS statistics package.

## Results

### Baseline characteristics

A total of 1541 young children (mean age: 11.2 ± 6.4 months, male: 50.7%) were included in this study. Detailed baseline characteristics of the participants were shown in **Table 1**.

**Table 1:** Demographic characteristics of the children and their ETS exposure condition (N = 1541).

Characteristics		All infants
Mean age (months)*		11.2 ± 6.4
Birth history*	Gestation weeks (weeks)	38.6 ± 1.4
	Birth weight (kg)	3.1 ± 0.4
BMI z score*		0.2 ± 1.3
Maternal age (yrs.)*		32.7 ± 4.6
Male gender (N, %)		782 (50.7)
Vaginal delivery (N, %)		1030 (66.8)
Have breastfeed ever (N, %)		1220 (79.2)
Number of young children (N, %)	2 months	477 (31.0)
in 3 age groups	12 months	522 (33.9)
	18 months	542 (35.2)
Have sibling (N, %)		748 (48.5)
Group care attendance ever (N, %)		197 (12.8)
Pet Keeping at home (N, %)		234 (15.2)
Living region (N, %)П	HK Island	338 (21.9)
	NT East	377 (24.5)
	KL	432 (28.0)
	NT West	394 (25.6)
Overcrowdingª (N, %)		96 (6.2)
Low income# (N, %)		447 (29.0)
Household ETS (N, %)	Current household ETS exposure	485 (31.5)
	Postnatal maternal smoking	53 (3.4)
	Prenatal maternal smokingσ	25 (1.6)
Number of household smoker (N, %)	0 household smoker	1052 (68.3)
	1 household smoker	381 (24.7)
	> 1 household smoker	108 (7.0)
Total cigarettes consumption amount	0 cigarette/ day	1052 (68.3)
of household smoker (s) (N, %)	1–20 cigarettes/ day	436 (28.3)
	>20 cigarettes/ day	53 (3.4)

*: Values are mean ± SD

ª: Household living area ≤ 5.5 m^2^ is regarded as overcrowding, in accordance with the guideline of the Hong Kong Housing Authority

#: Family income ≤20,000HKD is regarded as low income according to the HK statistic and census department 2014

σ: Maternal smoking during pregnancy

П: “HK Island” stands for “Hong Kong Island”; “NT East” stands for “New Territories East”; “KL” stands for “Kowloon”; “NT West” stands for “New Territories West”

The numbers of participants distributed in each gender, age and geographical region group were quite equal. Most of the children were born by vaginal delivery (66.8%) and had been breastfed (79.2%). About half (48.5%) of the young children had siblings. About 29% of the families had low household income and 6% had overcrowding of the household living areas.

### The exposure prevalence

The overall prevalence of household ETS exposure was 31.5%. Prevalence of prenatal and postnatal maternal smoking was 1.6% and 3.5% respectively. As for the exposure strata, the majority of the exposed young children (which accounted for 77.9% of the exposed ones) lived with 1 household smoker and the majority of the exposed young children (which accounted for 89.2% of the exposed ones) lived in household environment where the total daily cigarette consumption by the household smokers was 1–20 cigarettes.

### Risk factors of household ETS exposure

Characteristics associated with household ETS exposure are shown in **Table 2**.

**Table 2:** Univariate analysis of risk factors for household ETS exposure (N = 1541).

		Current household ETS exposure				Postnatal maternal smoking				Prenatal maternal smoking*			
		Exposure frequency	OR	95% CI	p value	Exposure frequency	OR	95% CI	p value	Exposure frequency	OR	95% CI	p value
Age group	2 months	27.3%	1			1.9%	1						
	12 months	33.0%	1.31	(1.00, 1.72)	**0.05**	4.2%	2.29	(1.04, 5.02)	0.23				
	18 months	33.8%	1.36	(1.04, 1.78)	**0.03**	4.1%	2.20	(1.00, 4.83)	0.33				
Gender	Boy	30.6%	1			4.1%	1		0.16				
	Girl	32.4%	1.09	(0.88, 1.35)	0.44	2.8%	1.50	(0.86, 2.62)	0.16				
Never breastfeed		41.4%	1.13	(1.07, 1.20)	**<0.001**	7.2%	3.06	(1.75, 5.35)	**<0.001**	3.7%	0.7	(0.32, 1.58)	0.43
Prenatal maternal smoking		80.0%	9.04	(3.37, 24.24)	**<0.001**	68.0%	87.4	(35.41, 215.52)	**<0.001**				
Have siblings (Not first parity)		33.9%	1.24	(1.0, 1.54)	0.06	4.0%	1.40	(0.81, 2.44)	0.26	1.6%	0.98	(0.45, 2.16)	1.00
Living region^П^	HK Island	22.8%	1			0.9%	1			0.6%	1		
	NT East	28.1%	1.33	(0.99, 1.86)	0.1	5.0%	5.93	(1.74, 20.21)	**0.004**	2.9%	5.05	(1.11, 22.95)	**0.04**
	KL	35.6%	1.88	(1.31, 2.59)	**<0.001**	3.7%	4.30	(1.24, 14.86)	0.21	1.4%	2.37	(0.48, 11.80)	0.29
	NT West	37.6%	2.04	(1.47, 2.83)	**<0.001**	3.8%	4.42	(1.27, 15.40)	**0.02**	1.5%	2.6	(0.52, 12.96)	0.24
Overcrowding		63.5%	4.20	(2.73, 6.46)	**<0.001**	6.3%	1.98	(0.83, 4.76)	0.14	2.1%	1.32	(0.31, 5.66)	0.71
Low household income		40.7%	1.79	(1.42, 2.26)	**<0.001**	4.9%	1.78	(1.02, 3.10)	**0.04**	3.1%	3.18	(1.43, 7.07)	**0.004**

*: Age and gender were not considered as potential risk factors of prenatal maternal smoking exposure

П: “HK Island” stands for “Hong Kong Island”; “NT East” stands for “New Territories East”; “KL” stands for “Kowloon”; “NT West” stands for “New Territories West”

### Current household ETS exposure

By univariate analysis (**Table 2**), factors associated with current household ETS exposure were: never breastfed; prenatal maternal smoking; living in Kowloon and New Territories (NT) West; overcrowding of household living place; lower household income; older in age. With multivariate analysis (**Table 3**), the results remained similar. According to our analysis results, model 2 had the biggest Nagelkerke R-square value of 0.098, followed by model 1 of 0.081 and model 3 of 0.077. Thus according to model 2, the independent factors associated with the current exposure were: never breastfed (AOR: 1.48, 95% CI: 1.13–1.93, p = 0.004); prenatal maternal smoking (AOR: 7.46, 95% CI: 2.73–20.39, p< 0.001); overcrowding of household living place (AOR:3.17, 95% CI: 2.02–4.96, P< 0.001); and lower household income (AOR: 1.34, 95% CI: 1.04–1.72, p = 0.02). Interestingly, we found some geographical differences of the ETS exposure distribution that young children residing in Kowloon (AOR: 1.66, 95% CI: 1.19–2.33, p = 0.003) and NT West (AOR: 1.54, 95% CI: 1.11–2.15, p = 0.01) were independently and significantly associated with household ETS exposure compared with children residing in Hong Kong Island.

**Table 3:** Multivariate analysis of risk factors for current household ETS exposure.

		Model 1 (N = 1541)			Model 2 (N = 1541)			Model 3 (N = 1516)		
		AOR	95% CI	p value	AOR	95% CI	p value	AOR	95% CI	p value
Never breastfeed		1.54	(1.18, 2.01)	**0.001**	1.48	(1.13, 1.93)	**0.004**	1.51	(1.16, 1.98)	**0.003**
Have siblings 6-18yrs		1.48	(1.12, 1.94)	**0.005**	1.44	(1.09, 1.90)	**0.01**	1.45	(1.10, 1.92)	0.09
Living region^П^	HK Island	1			1			1		
	NT East	1.21	(0.85, 1.70)	0.29	1.16	(0.81, 1.64)	0.42	1.15	(0.81, 1.64)	0.43
	KL	1.55	(1.11, 2.15)	**0.01**	1.54	(1.11, 2.15)	**0.01**	1.51	(1.08, 2.12)	**0.02**
	NT West	1.66	(1.19, 2.33)	**0.003**	1.66	(1.19, 2.33)	**0.003**	1.65	(1.72, 2.31)	**0.004**
Overcrowding		3.11	(1.99, 4.87)	**<0.001**	3.17	(2.02, 4.96)	**<0.001**	3.15	(2.01, 4.95)	**<0.001**
Low household income		1.38	(1.08, 1.77)	**0.01**	1.34	(1.04, 1.72)	**0.02**	1.32	(1.03, 1.70)	**0.03**
Pregnancy maternal smoking*					7.46	(2.73, 20.39)	**<0.001**			

*: Since the effect of prenatal maternal smoking on children’s ETS exposure condition could be difficult to fully ruled out, 3 different models were built: 1) by ignoring the effect of prenatal maternal smoking; 2) by adjusting to prenatal maternal smoking; 3) by excluding young children with prenatal maternal smoking

П: “HK Island” stands for “Hong Kong Island”; “NT East” stands for “New Territories East”; “KL” stands for “Kowloon”; “NT West” stands for “New Territories West”

### Postnatal maternal smoking

By univariate analysis (**Table 2**), the identified risk factors were never breastfed, prenatal maternal smoke, living in Kowloon and N.T West regions, and lower household income. According to our multivariate analysis results (**Table 4**), model 2 had the biggest Nagelkerke R-square value of 0.235, followed by model 1 of 0.076 and model 3 of 0.022. Thus, by multivariate analysis of model 2, the independent risk factors were never breastfed (AOR: 2.45, 95% CI: 1.29–4.64, p = 0.006) and prenatal maternal smoking (AOR: 75.9, 95% CI: 30.26–190.53, p< 0.001).

**Table 4:** Multivariate analysis of risk factors for postnatal maternal smoking.

Characteristics		Model 1 (N = 1541)			Model 2 (N = 1541)			Model 3 (N = 1516)		
		AOR	95% CI	p value	AOR	95% CI	p value	AOR	95% CI	p value
Never breastfeed		2.73	(1.55, 4.81)	**0.001**	2.45	(1.29, 4.64)	**0.006**	2.55	(1.29, 5.05)	**0.007**
Have siblings 6-18yrs		2.16	(1.20, 3.88)	**0.01**				** **		
Living region^П^	HK Island	1								
	NT East	5.15	(1.50, 17.65)	**0.009**						
	KL	3.62	(1.04, 12.63)	**0.04**						
	NT West	3.41	(0.97, 12.01)	0.06						
Prenatal maternal smoking					75.9	(30.26, 190.53)	**<0.001**			

П: “HK Island” stands for “Hong Kong Island”; “NT East” stands for “New Territories East”; “KL” stands for “Kowloon”; “NT West” stands for “New Territories West”

### Prenatal maternal smoking

By univariate analysis (**Table 2**), significant factors associated with prenatal maternal smoking found were living in Kowloon and N.T West regions and lower household income. With multivariate analysis, only lower household income (AOR: 3.18, 95% CI: 1.43–7.05, p<0.001) remained statistically significant in the adjusted model.

### Distribution of ETS exposure strata

We carried out this further analysis to explore the patterns of number of household smokers and total daily cigarette consumption by household smokers (**Table 5**).[18] The identified risk factors for higher exposure strata were never breastfed, prenatal maternal smoking, postnatal maternal smoking, having sibling, living in Kowloon and N.T West, overcrowding of household living place and lower household income.

**Table 5:** Household ETS exposure strata under different variables (N = 1541).

Characteristics		Number of Household smokers				Number of total daily cigarettes consumption of household smoker			
		0	1	>1		0	1–20	>20	
		N (Frequency)	N (Frequency)	N (Frequency)	P value	N (Frequency)	N (Frequency)	N (Frequency)	P value
Age group	2 months	347 (72.7%)	99 (20.8%)	31 (6.5%)	0.17	347 (72.7%)	117 (24.5%)	13 (2.7%)	0.08
	12 months	350 (67.0%)	137 (26.2%)	35 (6.7%)		350 (67.0%)	156 (29.9%)	16 (3.1%)	
	18 months	359 (66.2%)	141 (26.0%)	42 (7.7%)		359 (66.2%)	157 (29.0%)	26 (4.8%)	
Gender	Boy	543 (69.4%)	183 (23.4%)	56 (7.2%)	0.61	543 (69.4%)	211 (27.0%)	28 (3.6%)	0.71
	Girl	513 (67.6%)	194 (25.6%)	52 (6.9%)		513 (67.6%)	219 (28.9%)	27 (3.6%)	
Have breastfeed ever		868 (71.1%)	288 (23.6%)	64 (5.2%)	**<0.001**	868 (71.7%)	317 (26.0%)	35 (2.9%)	**<0.001**
Gestation> = 39 weeks		554 (65.9%)	219 (26.0%)	68 (8.1%)	**0.03**	554 (65.9%)	250 (29.7%)	37 (4.4%)	**0.02**
Prenatal maternal smoking		5 (20.0%)	7 (28.0%)	13 (52.0%)	**<0.001**	5 (20.0%)	13 (52.0%)	7 (28.0%)	**<0.001**
Postnatal maternal smoking		0 (0.0%)	10 (18.9%)	43 (81.1%)	**<0.001**	0 (0.0%)	39 (73.6%)	14 (26.4%)	**<0.001**
Not the first parity baby		493 (66.1%)	196 (26.3%)	57 (7.6%)	0.14	493 (66.1%)	218 (29.2%)	35 (4.7%)	**0.03**
Group care attendance		152 (77.2%)	35 (17.8%)	10 (5.1%)	**0.02**	152 (77.2%)	43 (21.8%)	2 (1.0%)	**0.008**
Living region^П^	HK Island	261 (77.2%)	69 (20.4%)	8 (2.4%)	**<0.001**	261 (77.2%)	71 (21.0%)	6 (1.8%)	**<0.001**
	NT East	271 (71.9%)	76 (20.2%)	38 (8.8%)		271 (71.9%)	94 (24.9%)	12 (3.2%)	
	KL	278 (64.4%)	116 (26.9%)	38 (8.8%)		278 (64.4%)	137 (31.7%)	17 (3.9%)	
	NT West	246 (62.4%)	116 (29.4%)	32 (8.1%)		246 (62.4%)	128 (32.5%)	20 (5.1%)	
Overcrowding		35 (36.5%)	40 (41.7%)	21 (21.9%)	**<0.001**	35 (36.5%)	51 (53.1%)	10 (10.4%)	**<0.001**
Low household income		265 (59.3%)	144 (32.2%)	38 (8.5%)	**<0.001**	265 (59.3%)	163 (36.5%)	19 (4.3%)	**<0.001**

П: “HK Island” stands for “Hong Kong Island”; “NT East” stands for “New Territories East”; “KL” stands for “Kowloon”; “NT West” stands for “New Territories West”

### Comparison with global and previous local data

The comparison of current study data with previous global and local data was summarized in **Table 6**. The overall prevalence of household ETS exposure 31.5% was lower than the 2004 global average figure 40% [4], and the 1997 local figure 42.1% [7].

**Table 6:** Comparison with previous global and local data.

Reference		Öberg M et al. [4]	Leung M et al. [7]	Mak YW et al. [29]	Current study
Region		Global	Hong Kong	Hong Kong	Hong Kong
Sampling period		2004	1997	1997	2014–2014
Participants		0–14 years old children	3, 9, and 18 months old children	4–5 years old children	2, 12, 18 months old children
Prevalence of ETS exposure (%)	Overall	40%	42.1%	NA, only studied children with smoking household	31.5%
	Prenatal maternal smoking	NA	4.8%	NA	1.6%
	Postnatal maternal smoking	NA	2.8%	NA	3.4%
Exposure strata by number of household smoker	1 household smoker	NA	NA	79.3% among families with smoking household	24.7%
	More than 1 household smoker	NA	NA	20.7% among families with smoking household	7.0%
Exposure strata by number of total cigarettes consumption amount of household smoker	1–20 cigarettes/ day	NA	NA	96.7% among all exposed children	28.3%
	More than 20 cigarettes/ day	NA	NA	3.3% among all exposed children	3.4%

## Discussion

Our study demonstrated the profile of household ETS exposure in Hong Kong healthy young children under 2 years of age. Prevalence of household ETS exposure in young children was 31.5%, prevalence of prenatal and postnatal maternal smoking was 1.6% and 3.4% respectively. Exposure was associated with never breastfed, prenatal maternal smoking, overcrowding of household living place and lower household income. Moreover, a geographical pattern of the exposure was found that young children residing in Kowloon and NT West were independently and significantly associated with household ETS exposure compared with children residing in Hong Kong Island. The identified risk factors of higher household ETS strata were quite similar with the identified risk factors of current household ETS exposure.

Comparison of the results from our study with previous study demonstrated that the overall prevalence of household ETS exposure in children younger than 2 years of age was lower compared with the 1997 data. This might reflect the success of the tobacco control milestones among very young children. In contrast, previous studies reported the increase in the exposure prevalence of indoor ETS exposure of school-aged children in recent decades.[9] Therefore, our findings may actually coincide with previous evidence demonstrating the protective effect of having young children at home against household smoking.[19]

For the exposure to postnatal and prenatal maternal smoking, the prevalence in our cohort was 3.4% and 1.6% respectively, which was similar with previous figures [7] and much lower than the western average figure 15% [20–21]. The prevalence of female smoking have been rising in Western Europe, Australia and the United States since the 20th century, while the figure in Hong Kong is consistently low.[22–23] The low prevalence might because of the efforts of the Hong Kong government and tobacco control advocates in raising tobacco tax, publishing legislation and anti- smoking campaigns etc. in the past decade.[23] Additionally, there is female-specific smoking cessation hotline service in Hong Kong, which is provided by The University of Hong Kong. Maternal smoking matters more than other household smoking despite the generally lower prevalence. Smoking mothers smoke more at home while other household smokers might smoke at work, additionally, mothers comparatively spend more time with the infants, and stay nearer with the baby, which let the exposure condition worse.[24] There is a lack of population-based smoking cessation interventions targeting Chinese woman smokers.[23] A recent local study demonstrated the effectiveness of one cessation counselling intervention which was given by trained counsellor according to the stage of readiness to quit.[25] Continued effort for female-specific tobacco control is needed. Scholars suggested that pregnancy is an opportune time to provide smoking cessation interventions for female smokers and their partners with an emphasis on the maintenance of post-partum smoking abstinence.[26] Smoking cessation strategies could be more early accepted by smokers at the health care circumstances [27], and being parents might further motivate the smoker to change.[28] There is a need to further develop female-specific smoking cessation intervention.

The construction of the exposure extent in our study was quite comparable to the data reported by *Mak YW* et al. [29], The construction did not significantly change over these two decades. It seems our tobacco control effort has not yet effectively relieved the exposure severity of those exposed young children.

The identified demographics or clinical risk factors can be grouped into three dimensionalities: (1) Lower SES which includes overcrowding of household living area and lower household income; (2) Unfavourable maternal behaviors which include no practice of breastfeeding ever and prenatal maternal smoking; (3) Geographical factor which was residing in Kowloon and NT West regions.

In our cohort, young children with lower family SES such as lower household income and overcrowding of household living area was associated with the ETS exposure. Similar observations were reported in several previous studies.[10, 24, 30–31] The underlying mechanism have not been fully investigated and explained. It could be complex and multifactorial. It is well recognized that lower family SES is linked to a series of disadvantaged health conditions and poor health-related behaviours such as tobacco use.[32–34] Families with lower SES could have lower parental education, disadvantaged growing environment for the children and inadequate parental health-related beliefs and behaviours.[35–36] The identified association between overcrowding and ETS exposure has particular implications as Hong Kong is a densely populated city. Overcrowding of the living area is not only a surrogate of lower SES of the family, it may also mean the children are more susceptible to the tobacco exposure because of the overcrowded environment.[10] Therefore, socially deprived children are at a higher risk of adverse long term health because of higher ETS exposure, worse health-related behaviours, and less access to medical service and optimal nutrition.[37]

Our study found that children with smoking mother had a much higher risk of household ETS exposure than those with non-smoking mother. This result was in line with the major finding of previous review study that maternal smoking is a strong predictor of children’s ETS exposure at home.[10] Smoking mother could have weaker consciousness of preventing their children from ETS exposure compared with non-smoking ones.[24] Thus, though the prevalence of maternal smoking is relatively low in Hong Kong, significant attention should be given to this group of smokers. It was found that negative emotions and stress were important factors in both smoking initiation and continued tobacco use among female smokers in Hong Kong.[23] Scholars highlighted that it is vital that healthcare professionals should focus on helping female smokers understand the health hazards of smoking, and counselling them about alternative strategies for coping with negative emotions and stress.[23]

No practice of breastfeeding ever was found to be associated with the exposure in a previous local study.[38] Previous research demonstrated that ETS may affect the maternal milk production, and the alternative explanation was lack of breastfeeding practice is an indicator of low SES, which is closely linked to smoking behaviours and less favourable health practice.[17]

Understanding the geographical varying pattern of one disease or one health-related condition could be very significant for the profile draw and related health promotion planning.[39–40] We found the exposure distribution was not equal among the 4 main regions in Hong Kong. The exposure risk was especially high in Kowloon and NT West regions, the need of ETS exposure control in young children of these regions should be highlighted. The reasons for this geographical pattern are not fully understood and could be complex. One important potential reason is the variation in the SES profile among these four regions [41–42], which in other words, the living region may be a surrogate of family SES, and therefore associated with ETS exposure. A classical English study found the influence of community levels of smoking within towns on passive exposure status of children aged 5–7 years old.[43] However our local data of region-specific smoking prevalence were not available to confirm such postulation.

Our study was territory representative, and we provided precious information on children under 2 years of age. However, there remained some limitations. First, for ETS exposure measures, objective measurement such as urinary cotinine and salivary cotinine was not adopted, which could be a shared limitation with the previous local study.[7] Moreover, socially desirability bias may reduce the validity of maternal self-reporting on smoking habit. Some groups of people are more reluctant than others to disclose their smoking status and exposure to tobacco, especially pregnant women and parents of young children, whose smoking is often regarded as socially unacceptable. However the general validity of parental self-reported smoking condition in Hong Kong has been proven to be valid and reliable previously.[44]

Second, the questionnaire we used to collect data was not validated. There is no validated international tool for ETS exposure profile study so far, and our study methods especially the variable measures are similar with previous studies [7, 15].

Third, as the current study used secondary data from our previous surveillance study, which was designed with different objectives, it might render non-specific results under the problem with some confounding factors not evaluated. Information about parental education and occupation was not collected, while these two factors could be important aspects of family SES. The type of tobacco product consumption was not recorded in our questionnaire, however cigarettes were still reported to be the dominant type of tobacco consumption among Hong Kong smokers according to the latest Hong Kong Government’s Thematic Household Survey Report (daily smokers by consumed products daily: cigarettes- 99.1% of all daily smokers; electronic cigarettes- 0.9% of all daily smokers; other types-0.6% of all daily smokers).[45] Future studies should be done to evaluate children’s exposure to different kinds of tobacco products. And data of more years, with seasonal trend is warranted to gain a deeper understanding of the epidemiology profile of ETS exposure among young children.

Finally, the previous surveillance study is limited by a moderate response rate about 60%, the major reason was parental refusal to consent for nasopharyngeal swab.

As household ETS exposure profile has never been comprehensively drawn in Hong Kong young children previously, and the data of our previous surveillance study could be regarded as representative with its community based nature and territory-wide recruitment. Thus, despite the limitations, current study could still provide precious information of household ETS exposure profile in Hong Kong young children.

## Conclusions

Findings of this study add to our knowledge that household ETS exposure is prevalent among Hong Kong young children, especially those with smoking mother and those in socially deprived families. More resources should be put to reduce the ETS exposure in our young children, particularly those from disadvantaged families. Early identification, early intervention.
